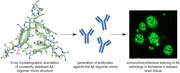# Development of Alzheimer’s disease immunotherapies using conformationally constrained β‐strands and β‐hairpins from Aβ and tau

**DOI:** 10.1002/alz.094742

**Published:** 2025-01-09

**Authors:** Adam Kreutzer, Jason Zhu, Jovanna Plancarte, Sarah Ruttenberg, James Nowick

**Affiliations:** ^1^ University of California Irvine, Irvine, CA USA

## Abstract

**Background:**

Peptide antigens that mimic disease‐related conformations of Aβ and tau were designed and created, and antibodies against these peptide antigens were generated and characterized.

**Method:**

The peptide antigens were designed to mimic β‐strands formed by Aβ and tau in the cryo‐EM structures of the Alzheimer’s disease brain‐derived fibrils. Peptide antigens were also designed to mimic β‐hairpins of Aβ and oligomers formed by the β‐hairpins. These β‐strand and β‐hairpin antigens were then used to generate antibodies in rodents. *Ex vivo* immunohistochemical and biochemical studies in Alzheimer’s disease brain‐tissue and cell‐based studies were performed to characterize the antibodies.

**Result:**

The antibodies raised against the Aβ‐derived peptide antigens recognized Aβ plaques in Alzheimer’s disease brain tissue and in 5xFAD mouse brains and protected iPSC‐derived human neurons from Aβ toxicity. Biochemical characterization of these Aβ‐derived antibodies showed that the antibodies exhibit some selectivity for aggregated forms of Aβ. The antibodies raised against the tau‐derived peptide antigens recognized tau neurofibrillary tangles in Alzheimer’s disease brain tissue and inhibited tau seeding in a cell‐based tau biosensor assay.

**Conclusion:**

Antibodies generated against conformationally constrained β‐strand and β‐hairpin antigens designed to mimic disease‐related conformations of Aβ and tau exhibit characteristics beneficial for immunotherapy, such as recognition of Aβ and tau pathology, and protection against Aβ cytotoxicity and inhibition of tau seeding.